# Higher Trophic Status Leads to More Diverse and Divergent Microeukaryote Communities Over Time in Urban Lakes From the Greater Paris (France)

**DOI:** 10.1111/1758-2229.70355

**Published:** 2026-04-28

**Authors:** Sébastien Duperron, Pierre Foucault, Amaury Le Vern, Midoli Goto, Charlotte Duval, Benjamin Marie, Sahima Hamlaoui, Sébastien Halary, Dominique Lamy, Emilie Lance, Marc Troussellier, Cécile Bernard, Ludwig Jardillier, Julie Leloup

**Affiliations:** ^1^ Muséum National d'Histoire Naturelle, UMR 7245 CNRS‐MNHN, Molécules de Communication et Adaptation des Microorganismes (MCAM) Paris France; ^2^ Sorbonne Université, UMR 7618 CNRS‐INRA‐IRD‐Univ. Paris Cité‐UPEC, Institut d'Écologie et des Sciences de l'Environnement de Paris (iEES‐Paris) Paris France; ^3^ Marine Biodiversity, Exploitation and Conservation (MARBEC) Univ. Montpellier‐CNRS‐Ifremer‐IRD Montpellier France; ^4^ Université de Reims, UMR‐I 02, Stress environnementaux et biosurveillance des milieux aquatiques (SEBIO) Reims France; ^5^ Université Paris‐Saclay, CNRS, AgroParisTech, Écologie Société et Évolution Gif‐sur‐Yvette France

**Keywords:** eutrophication, freshwater, microbial networks, phytoplankton, protists, time‐series, trophic mode

## Abstract

Effects of trophic status on lake microeukaryote community dynamics remain underexplored. Spanning oligotrophic to hypereutrophic conditions, peri‐urban lakes located in the Greater Paris region (France) offer unique opportunities to compare these dynamics along a eutrophication gradient. Here, community composition was characterised using 18S rRNA gene metabarcoding, and analyzed in the context of environmental parameters and chlorophyll *a*‐derived trophic status throughout an 18‐month sampling period. Microeukaryote assemblages were dominated by Cryptophyceae, Spirotrichea, Chrysophyceae, and Chlorophyceae, with mixotrophic and heterotrophic taxa dominating across all lakes. Taxa richness peaked at intermediate trophic levels, consistent with the intermediate disturbance hypothesis. Beta‐diversity and network analyses revealed increasing community modularity, reduced connectivity, and enhanced temporal variability with higher trophic status. In lakes reaching the hypereutrophic status, communities diverged progressively over time. Conversely, oligo‐ to mesotrophic lakes maintained more connected and stable assemblages. These findings demonstrate that eutrophication fosters more diverse and increasingly divergent microeukaryote communities, underscoring its role as a central driver of microbial community restructuring in urban freshwater systems.

## Graphical abstract

Microeukaryote dynamics across a eutrophication gradient in Greater Paris region lakes showed richness peaking at intermediate trophic levels and communities becoming more modular, variable, and less connected with higher trophic status compared to oligo‐to‐mesotrophic lakes.

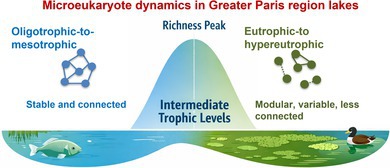

## 1 Introduction

Of the 300 million lakes on Earth, approximately 90% are considered small water bodies (less than 1 km^2^; Downing et al. 2006; Pi et al. 2022). Small lakes and ponds, being natural or artificial, harbour and sustain a large biodiversity, play key roles in the continental carbon cycle, at the interface between terrestrial ecosystems and the atmosphere, and act as final collection points for watersheds (Pi et al. 2022; Strayer and Dudgeon 2010). They exhibit rapid responses to localised anthropogenic disturbances, previously evaluated in particular in peri‐urban lakes and ponds (Catherine et al. 2008; Escalas et al. 2019; Kraemer et al. 2020). They are considered as suitable environmental sentinels for assessing the overall health of the surrounding ecosystem (Williamson et al. 2009). Lakes also offer a wide range of ecosystem services including essential supplies of drinking water, crop irrigation, and opportunities for recreational activities. Over the past decades, lakes have experienced significant impacts from human activities (e.g., agriculture, urbanisation), invasive species, increasing surface temperatures and heatwaves, and are considered under threat (O'Reilly et al. 2015; Reid et al. 2019; Williamson et al. 2009; Woolway et al. 2022). These pressures, combined with natural variations, have exacerbated eutrophication, resulting in widespread disruptions in ecosystem functioning, including increased microalgal and cyanobacterial blooms, deoxygenation and salinisation (Dugan et al. 2020; Jane et al. 2021). This is particularly pronounced in smaller lakes which are fragmented habitats with limited buffering capacities, and contribute to relatively high amounts of carbon emissions relative to their area (Holgerson and Raymond 2016; Mitsi et al. 2023). Numerous studies have investigated the impact of anthropogenic disturbances on lake prokaryotic communities, identifying global trends, such as highly variable taxa compositions especially under the influence of temperature and nutrient contents (Foucault et al. 2025; Garner et al. 2023; Shen et al. 2019). However, the main ecological functions overall tend to be conserved due to high functional redundancy among taxa (Allison and Martiny 2008; Foucault et al. 2025; Louca et al. 2018).

Lakes also harbour a rich, diverse and abundant community of mainly microbial eukaryotes (Debroas et al. 2017; Escalas et al. 2019; Simon et al. 2015). These have been less investigated despite their major roles in ecosystem functioning, as primary producers, predators, parasites and recyclers of organic matter (Adl et al. 2019; Garner et al. 2022; Itoïz et al. 2022; Monjot et al. 2023; Sommeria‐Klein et al. 2021). Recent one‐shot explorations at the regional and continental scales have for example revealed how land‐use, edaphic conditions and trophic status shape microeukaryotes assemblages (Bock et al. 2020; Garner et al. 2022). On the other hand, even within a limited area, small peri‐urban lakes can also display markedly different trophic status owing to the patchy distribution of local anthropic impacts. These offer a unique opportunity to test the impact of trophic status on community compositions by minimising the effect of confounding factors and facilitate time‐series sampling to evaluate community composition variability. With hundreds of mostly small lakes and reservoirs at the scale of the Île‐de‐France region (Catherine et al. 2008), Paris is an exceptional natural laboratory to explore these relationships. A previous study pointed out that trophic status was a main driver of bacterial community structure during a summer period, that the functional potential was stable and mostly shared among lakes, and that community heterogeneity increased with higher trophic levels (Foucault et al. 2025).

In the present study, we determined the effect of eutrophication levels on the composition of microeukaryote communities and their associated trophic mode (phototroph, mixotroph or heterotroph) based on an 18‐month survey of communities' compositions on nine lakes displaying different levels of eutrophication, all located within a 70‐km distance around Paris. We hypothesise that (i) the trophic status has an impact on the microeukaryote communities, but their temporal variations are comparable because of the close proximity among lakes, (ii) that community variability will decrease under higher trophic status owing to the dominance of a limited set of taxa; and that (iii) different trophic groups respond differently, with phototrophs being more sensitive than mixotrophs and heterotrophs to local conditions.

## 2 Material and Methods

### 2.1 Sampling

Nine peri‐urban lakes were monitored, namely Jablines (JAB), Vaires‐sur Marne (VSM), Cergy large (CER‐L), Cergy small (CER‐S), Créteil (CRE), Bois‐le‐Roi (BLR), La Grande Paroisse (LGP), Champs‐sur‐Marne (CSM), and Verneuil‐sur‐Seine (VSS). Lakes are located within a ~70 km radius around Paris (France; Figure S1 and Table S1 for coordinates) and were chosen owing to their similar area and depth (7.3–91.0 ha, 3.5–10 m). All are former sand and gravel quarries converted into leisure centers between the 1960s and the 1980s (Catherine et al. 2008, 2010; Escalas et al. 2019). Lakes were sampled monthly from June 2021 to December 2022. Lack of access prevented sampling on all lakes in December 2021, and at CER‐L in June 2021. Overall, a total of 18 time‐points were thus sampled from each lake (17 for CER‐L).

For each lake and date, the water column was sampled at three mid‐lake locations (labelled W1, W2 and W3) to account for spatial heterogeneity (exact coordinates of sampling sites can be found in Figure S1 from Foucault et al. 2025). For each water column, 5 L were sampled using a Niskin bottle (WILDCO, USA) at three different depths (~0.5 m below surface, mid‐depth and ~0.5 m above the lake bottom) and pooled together in equal volumes (depth‐integrated sample). Samples were processed on‐site within 1 h post‐recovery. A total of 483 samples were collected and analyzed.

### 2.2 Chlorophyll a Concentration and Phytoplankton Composition

**FIGURE 1:**
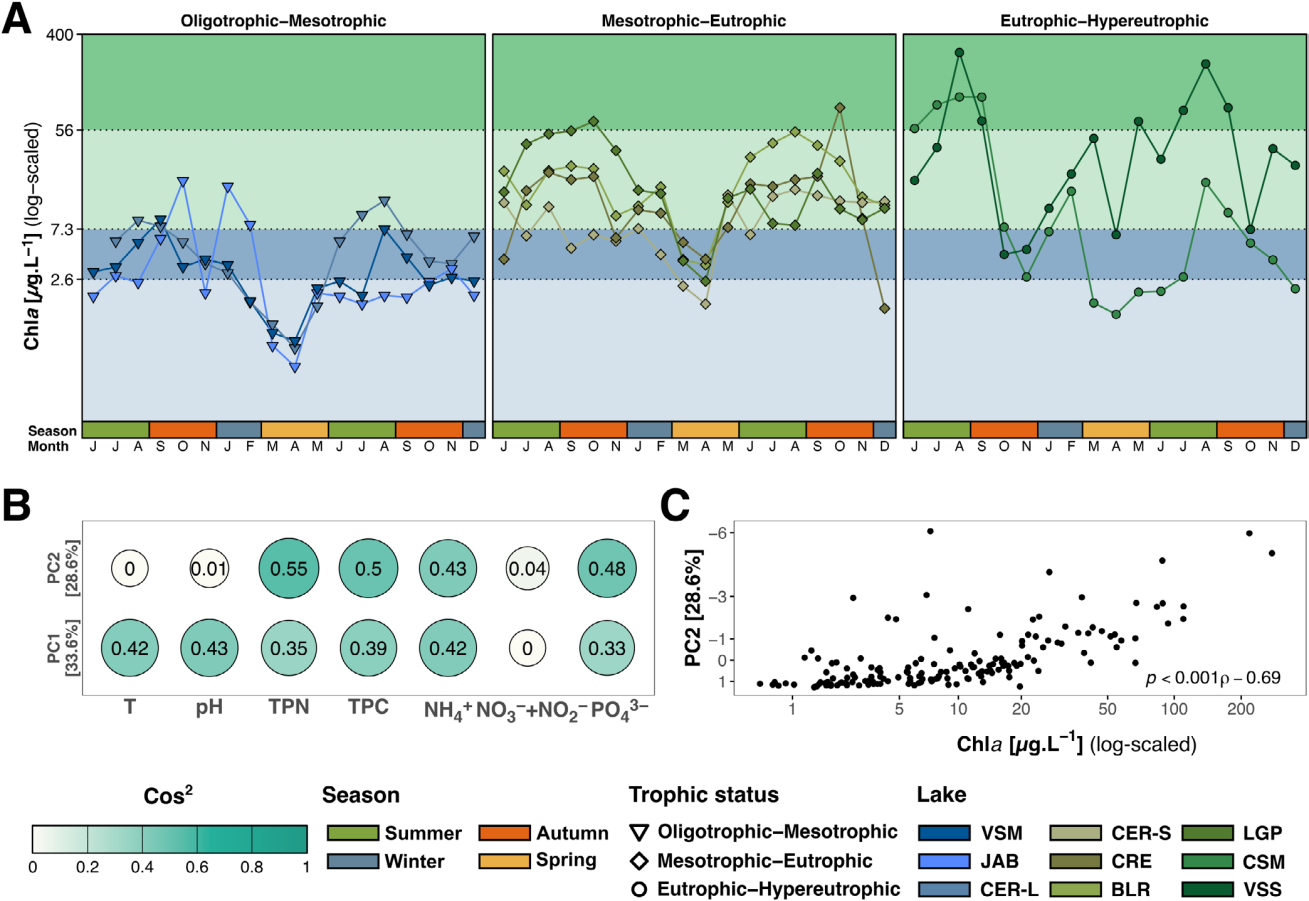
Temporal dynamics of Chl*a* concentrations (A) Average Chl*a* levels (*n* = 3 per month, except for VSM in July 2021 *n* = 2) from June 2021 to December 2022. Background colours correspond to Carlson's TSI trophic states: Oligotrophic (light blue), mesotrophic (dark blue), eutrophic (light green), hypereutrophic (dark green, see material and methods). The y‐axis is log‐scaled, the x‐axis corresponds to the month (i.e., initial of each month, and colour bars correspond to seasons). (B) Correlation (Cos^2^ values) of each feature with the two first axes of the PCA based on scaled and centered water parameters except Chl*a* concentration. (C) Relationship between the PC2 axis coordinates and the Chl*a* concentration. The x‐axis (Chl*a* concentration) was log‐scaled. Spearman correlation statistics are displayed.

The chlorophyll *a* (Chl*a*) concentration was measured as a proxy of phytoplankton biomass from 500 mL filtered raw‐water (0.7‐μm, GF/C, Whatman, UK) in triplicate (Table S2), by spectrophotometry (Cary 60 UV–Vis, Agilent, USA, Yéprémian et al. 2016). At each timepoint, the trophic status of the lakes was determined based on Chl*a* concentration ranges of the Carlson's trophic state index (CSI, Carlson 1977): oligotrophic (< 2.6 μg·L^−1^), mesotrophic (2.6–7.3 μg·L^−1^), eutrophic (7.3–56 μg·L^−1^) and hypereutrophic (> 56 μg·L^−1^; Figure 1A). Phytoplankton taxa were identified based on morphology from lugol‐fixed unfiltered water under an inverted microscope (NIKON Eclipse TS100, Japan), using the Utermöhl method (AFNOR 15204 standard, 200 to 400 individuals). The biovolume of each taxon was estimated by multiplying its cell counts by its associated cell biovolume values based on previous reports (Escalas et al. 2019; Maloufi et al. 2016) that used geometric shape assumptions methods (Hillebrand et al. 1999; Liu and Sun 2003). For taxa that were not in these reports, cell biovolumes were directly extracted from the 2017 HELCOM Phytoplankton Expert Group database (Olenina 2012).

### 2.3 Water Physico‐Chemical Parameters

Temperature and pH were measured on site (KS‐2 MultiLine probe, WTW, USA). Raw water samples were pre‐filtered on 50‐μm mesh to remove large particles (e.g., leaves, larvae, largest microbial eukaryotes). Then, water was filtered onto 0.22‐μm PES membranes (Millipore Express, Germany). Eluates were collected in duplicate (12 mL) in polyethylene tubes for nutrient analyses, with an acidification step (three droplets of 3% HNO_3_ solution) for orthophosphate analysis (PO_4_^3−^ ions). Dissolved mineral nitrogen (NH_4_^+^, NO_3_^−^ and NO_2_^−^ ions) and PO_4_^3−^ concentrations were determined as described by Holmes et al. (1999). For total particulate carbon and nitrogen concentration (TPC and TPC), 1 L of raw‐water was filtered onto 0.3‐μm pre‐combusted filters in duplicates (Sterlitec, USA). Filters and eluates were stored at −20°C. Total particulate carbon and nitrogen concentration were determined using a CHN Elemental Analyser (NA1500 Series 2, Fisons, UK) and normalised by sampled volume (Table S3).

### 2.4 DNA Extraction

For each depth‐integrated water column, 150 to 2000 mL (depending on the lake and the month) of pre‐filtered water were filtered onto 0.22‐μm PES membranes. Filters were flash‐frozen in liquid nitrogen for DNA conservation. Total DNA was extracted (483 filtered water samples) using the PowerLyzer PowerSoil DNA extraction kit (QIAGEN, Germany), after a bead‐beating step (FastPrep‐24 5G, MP Biomedical): 5 × 30 s cycles (8 m.s^−1^). Two extraction blanks and two positive controls were incorporated into the sequencing analyses. Twelve samples were re‐sequenced during the second Illumina run to intercalibrate the two sequencing runs.

### 2.5 18S rRNA‐Encoding Gene Amplicon Sequence Analysis

The V4 hypervariable region of the eukaryotic 18S rRNA‐encoding gene was amplified using primers EUK581‐F (5′‐GCAGTTAAAAAGCTCGTAGT‐ 3′) and EUK1134‐R (5′‐TTTAAGTTTCAGCCTTGCG‐ 3′) (Carnegie et al. 2003; Pawlowski et al. 2012). These primers are reportedly biased against metazoans and fungi, leading to potential underestimation of their abundances (Bower et al. 2004). The following program was used: initial denaturation (94°C, 3 min); 35 cycles (94°C, 45 s; 55°C, 60 s; 72°C, 90 s); final elongation (72°C, 10 min). Amplicons were sequenced on an Illumina MiSeq 300 × 2 bp platform (GenoToul, France). A total of 31,200,822 raw reads was obtained.

Sequences were analyzed using the QIIME2 pipeline (version 2024.5; Bolyen et al. 2019). Amplicon Sequence Variants (ASVs) were obtained using the DADA2 plugin (v1.30.0; Callahan et al. 2016): reverse reads were trimmed at 245 bp due to lower phred‐scores, while the forward read was not trimmed. The expected error rate was set at 3, minimum overlap to 4 bp and chimeras were discarded. ASVs were then affiliated taxonomically using the *sklearn* algorithm trained on the PR^2^ database (Guillou et al. 2013) (5.1.0ssu). ASVs affiliated to Bacteria, Metazoa, Mitochondria, Chloroplast, Streptophyta, plastids, nucleomorphs or unassigned were discarded. The remaining dataset contained 14,981,309 high‐quality assembled non‐chimeric eukaryote reads (31,017 ± 13,420 per sample). Rarefaction was performed at 12,287 reads (lowest appropriate sample sequencing depth, after removing four samples due to low number of reads, namely CRJ‐P‐W3, TRI‐B‐W1, VAI‐O‐W1, CHA‐N‐W1; Table S1). Sample VSM_B_W1 was also discarded from all analyses based on the aberrant measured Chl*a* concentration (Table S2). The analysis yielded 19,714 unique ASVs. Trophic modes, namely phototroph, mixotroph, phagotroph and parasite, were assigned at the 4th taxonomic rank (PR^2^ Class level; Table S4) based on the PR^2^ database (version 5.1.0; Guillou et al. 2013) and additional literature (Adl et al. 2019; Monjot et al. 2023; Singer et al. 2021; Sommeria‐Klein et al. 2021). Although it may not be fully accurate since intra‐taxon variability exists, this is the lowest taxonomic rank with reliable assignation for most ASVs, allowing inter‐lakes comparisons.

### 2.6 Statistical Analysis

Analyses were run on Rstudio (v4.4.3, The R Core team 2022). The Chl*a* concentrations were computed by lake and month. A PCA was performed on environmental parameters (centered and scaled values) excluding Chl*a* values. The contribution of each parameter on the two first components were displayed as cos2 values. The correlation between the two first PCA component coordinates and the Chl*a* values was assessed by a Spearman correlation test (Rho coefficient (*ρ*), cor.test, Stats Rbase package v4.4.3).

Alpha‐diversity indexes (ASV richness and Shannon diversity) were computed using Phyloseq (v1.50.0; McMurdie and Holmes 2013). Principal Coordinate Analyses (PCoA) were performed based on Bray‐Curtis (BC) distances using Vegan (Oksanen et al. 2022). The explanatory power of the ‘lake identity’, ‘season’ and their interaction term were tested using PERMANOVA (*adonis2* from Vegan).

A time lag analysis (TLA) was run individually based on date‐to‐date pairwise Bray‐Curtis dissimilarities normalised by the number of days elapsed between respective sampling months. For each dataset, linear and polynomial models (2nd to 5th degree) were fitted. Model fit was evaluated using the Akaike Information Criterion (functions *AIC()* and *lm()* from Lme4 (v1.1‐37); (Bates et al. 2015) and LmerTest (v3.1‐3); Kuznetsova et al. 2017).

Changes in community composition over time in each lake were estimated using the MOTA (Multivariate Omics Trajectory Analysis) method (Foucault et al. 2022). Briefly, a PCoA with minimum number of axes necessary to account for more than 90% of explained variance was computed. Then, for each lake, “month” centroids were generated by averaging coordinates from these PCoA axes of the three water columns sampled on a given month. Then, distances between centroids (Euclidian norm) from consecutive time points are summed to compute the length of the total trajectory travelled by monthly centroids during 17 months (starting from July 2021, as CER‐L was not sampled in June 2021). Total lengths of lake trajectory could be compared, as there were extracted from the same PCoA axis, and reflect the amount of changes chronologically from the first to last time point. The relationship between MOTA trajectory lengths and the 18‐month Chl*a* mean of each lake was assessed by a Spearman correlation test (Rho coefficient (*ρ*), cor.test, Stats Rbase package v4.4.3).

For each lake, a co‐occurrence network was built using only the abundant core ASVs of this lake. Abundant core ASVs were defined as that represent at least 1% of the reads in at least one sample from this lake. Networks were built using NetCoMi (v1.2.0; method: SparCC, min. corr. 0.6, Peschel et al. 2021) and igraph (v2.1.4) and network statistics were computed. The percentage of connected nodes assigned to each trophic mode as well as the percentage of modules with one, two, three or the four trophic modes and the trophic mode richness of each module were also calculated.

## 3 Results

### 3.1 Temporal Variations of Lake Trophic Status

Temporal variations in trophic status exhibited contrasting patterns across the nine lakes (Figure 1A; Table S2). During most of the 18 months, lakes VSM, JAB, and CER‐L maintained a relatively stable low Chl*a* level, within the oligotrophic‐to‐mesotrophic range according to the CSI index (average 3.35 ± 2.70, 4.33 ± 5.57, and 5.18 ± 4.51 μg·L^−1^, Figure 1A), and were thus classified as “oligotrophic‐to‐mesotrophic”. Lakes CER‐S, CRE, BLR, and LGP displayed intermediate Chl*a* levels and temporal fluctuations, mostly within the mesotrophic‐to‐eutrophic range and were thus classified as “mesotrophic‐to‐eutrophic”. Lakes VSS and CSM showed a larger range of values (0.6 to 134 and 3 to 374 μg·L^−1^, respectively) and reached the hypereutrophic status during at least 4 months. They were thus classified as “eutrophic‐to‐hypereutrophic”.

A PCA was performed on water environmental parameters (T, pH, TPC, TPN, PO_4_^3−^, NH_4_^+^, NO_3_^−^ + NO_2_^−^; Figure S2; Table S3) revealing that samples were segregated by pH and T on the first axis (33.6% explained variance; cos^2^ 0.43 and 0.42; Figure 1B) while axis 2 (28.6% explained variance) was correlated to nutrients (TPC, TPN, PO_4_^3−^, NH_4_^+^; cos^2^ 0.55, 0.5, 0.48 and 0.43; Figure 1C). Coordinates on both PCA axis were correlated to Chl*a* concentrations (SPEARMAN, *p* < 0.001, *ρ* 0.4 and 0.69; Figure 1C; Table S5), confirming that Chl*a* concentrations are a good proxy for the overall nutrient status of the lake.

Based on microscopy identification, the phytoplankton community was dominated by eukaryotes throughout the survey period (18‐month average: 80.8% ± 28.7%; Figure S3) in all lakes, except for BLR. Four major groups were the most abundant: Diatoms, Cryptophyceae, Dinophyceae and Cyanobacteria (Figure S3; Table S6). In the Lake BLR, Cyanobacteria were dominant (58.8% ± 27.1%; 18‐month average).

### 3.2 Microeukaryote Community Composition

**FIGURE 2:**
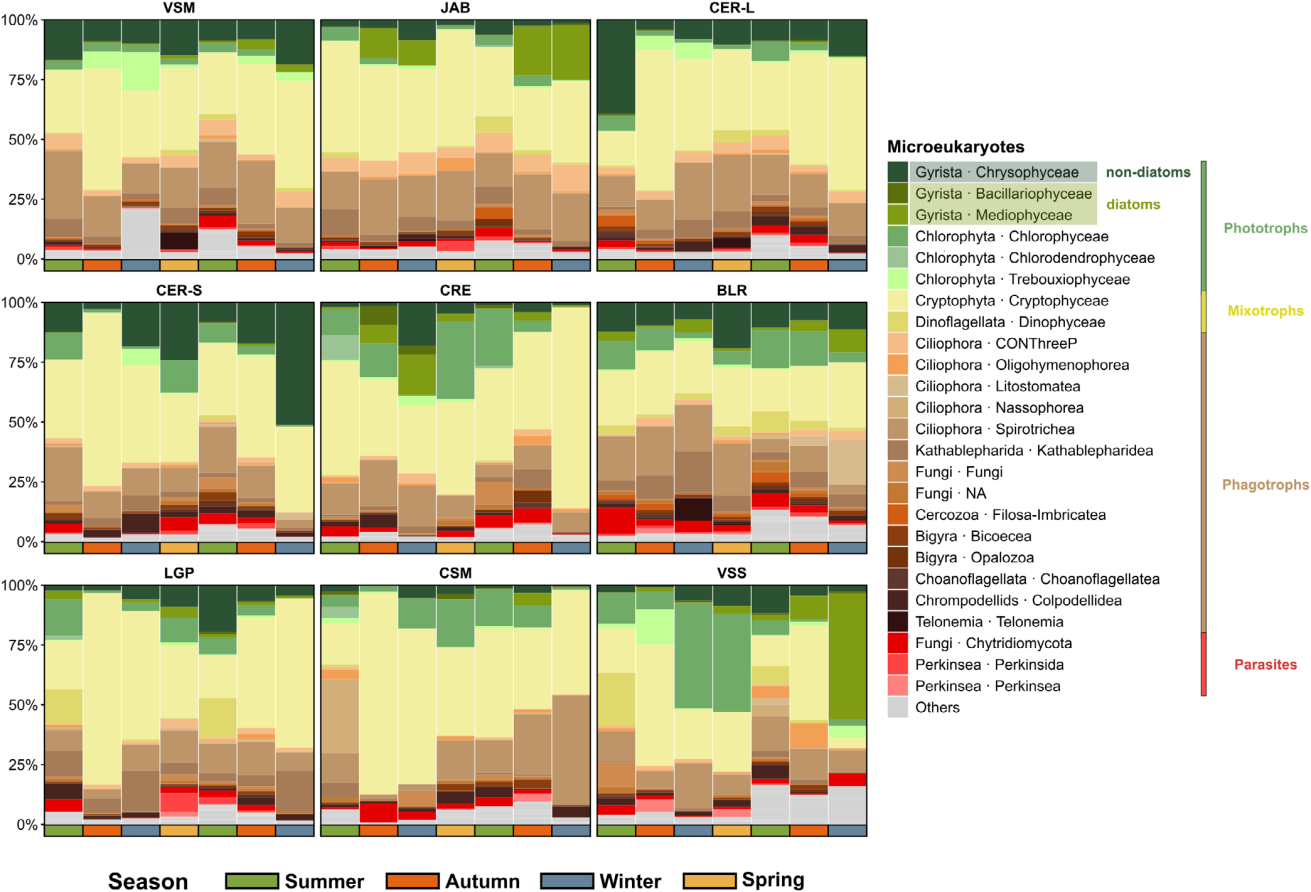
Taxonomic composition of microeukaryote communities. Median proportion of the total ASV reads per season. The 25 most abundant classes are coloured according to their potential trophic mode according to the database PR^2^ v5.0 (phototrophs, mixotrophs, phagotrophs and parasites). On the x‐axis, the colour bars correspond to seasons. Lake panels are ordered according to increasing 18‐month averaged Chl*a* concentration (from left to right, then from top to bottom).

Microeukaryote communities (0.22–50 μm size fraction) were characterised based on 18S rRNA ASVs metabarcoding, and each ASV was associated to one of four trophic modes: phototrophs, mixotrophs, phagotrophs and parasites (Table S7). Communities were dominated by four classes: Cryptophyceae (Cryptophyta), Spirotrichea (Ciliophora), Chrysophyceae (Gyrista) and Chlorophyceae (Chlorophyta) for all lakes, seasons and months (Figures 2 and S4; Table S8). Phagotrophs and parasites represented 30.1% and 21.4% of all ASVs, and 30.9% and 6.2% of all reads, respectively. Phototrophs accounted for 29.7% of all ASVs and 23.0% of all reads. Finally, mixotrophs only accounted for 6.6% of all ASVs, but represented 39.0% of all reads. The most prevalent ASV, identified as the mixotroph *Cryptomonas curvata* (Cryptophyta), alone accounted for 18.9% of total reads in the whole dataset, emphasising the abundance and ubiquity of certain taxa.

All the microeukaryote communities exhibited similar temporal patterns in alpha diversity indexes, with higher ASV richness observed during the summer (Figure S5). Values ranged from 116 ± 72 ASVs in the winter of 2021 to 304 ± 99 in the summer of 2022 (Figure S5A). Similar seasonal patterns were observed across the different trophic modes (Figure S5C–F). Lakes with the lowest (JAB, oligotrophic‐to‐mesotrophic) and highest (VSS, eutrophic‐to‐hypereutrophic) average Chl*a* concentrations both exhibited lower ASV richness than lakes displaying intermediate Chl*a* levels.

**FIGURE 3:**
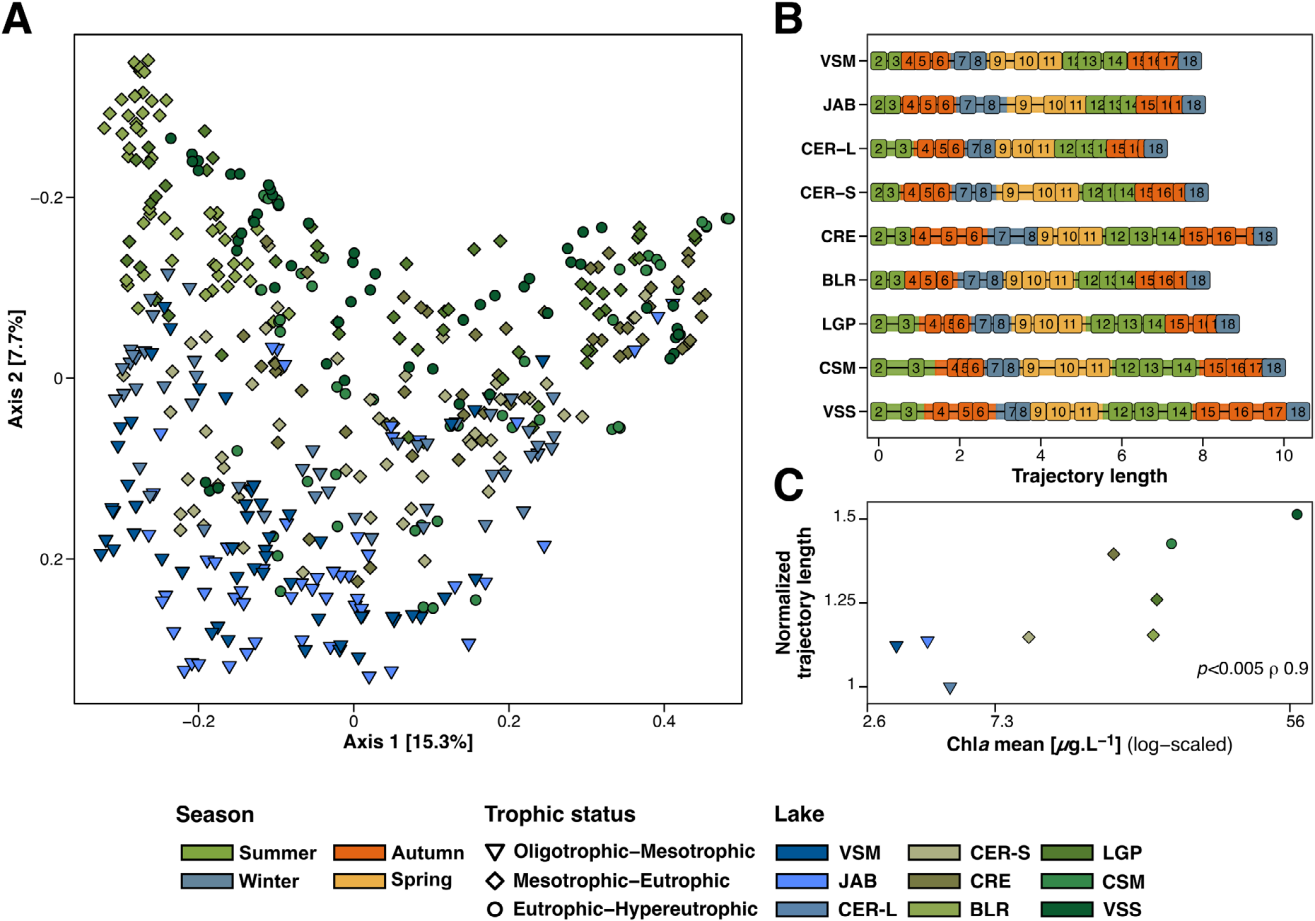
Microeukaryote communities composition and MOTA trajectory length (A) PCoA plot based on Bray–Curtis dissimilarity of microeukaryote communities. Individual panels, constructed with the same coordinate's set for each lake, are displayed in Figure S6, in order to illustrate the impact of seasons. (B) MOTA month‐to‐month cumulative trajectories lengths of the communities (BC dissimilarity). Trajectories were calculated on the first 56 axes (90.26% of the total explained variance). The number corresponds to the sampling month (i.e., 2 for January and 12 for December). The lakes (y‐axis) are ordered according to increasing 18‐month averaged Chl*a* concentration (from top to bottom). (C) Relationship between the trajectory length in a given lake and its 18‐month averaged Chl*a* concentration. All trajectory lengths were normalised to the shortest. The x‐axis (Chl*a* concentration) was log‐scaled. Spearman rho statistics is displayed.

Community structure comparisons (Bray–Curtis dissimilarity) indicated significant segregation by lake (*p* < 0.001 and *R* ^2^ = 0.21, Figure 3A), then, to a lower extent, by season (*p* < 0.001 and *R* ^2^ = 0.07; Table S9). However, the data corresponding to the different lakes overlapped to some extent (Figures 3A and S6). The microeukaryote communities of the different lakes displayed varying temporal dynamics (PERMANOVA; lake: season interaction; *p* < 0.001 and *R* ^2^ = 0.16; Table S9): a significant effect of trophic status was observed among the communities of the oligotrophic‐to‐mesotrophic (VSM, JAB and CER‐L), mesotrophic‐to‐eutrophic (CER‐S, CRE, BLR, LGP) and the eutrophic‐to‐hypereutrophic (CSM and VSS) lakes (*p* < 0.001 and *R* ^2^ = 0.08; Figure 3A, Table S9). Specifically, the trophic status of lake CSM transitioned over time from hypereutrophic (summer and early autumn of 2021) to mesotrophic status (2022), with occasional shifts to eutrophic and oligotrophic levels (Figure 1A). This major change is reflected on its associated eukaryote community compositions, with limited overlap between polygons of summers 2021 and 2022, and no overlap between autumns 2021 and 2022 (Figure S6). Concerning the lake BLR, its polygons displayed very limited overlap with those from other lakes, indicating different communities (Figure S6). Besides, the BLR polygons corresponding to each season were very small and largely overlapping with one another compared to all other lakes, suggesting a stable community composition in this lake.

When focusing on mixotroph, phototroph, and phagotroph trophic modes, community compositions were also affected by both lake and season (PERMANOVA; *p* < 0.001, *R* ^2^ values ranging from 0.16 to 0.25 for lakes, 0.04 to 0.08 for season; Figure S7; Table S9). For parasites, the effect of lake identity was of lesser importance (*p* < 0.001, *R* ^2^ values 0.09 for lakes and 0.03 for season; Figure S7; Table S9). For all trophic modes, the seasonal effect on the community composition varied greatly among lakes (*p* < 0.001, *R* ^2^ values ranging from 0.15 to 0.17 for lake and season interactions; Figure S7; Table S9).

The amplitude of changes in community composition over time was estimated by summing the distances separating each centroid (1 centroid per month) throughout the 18 months using the MOTA method. Shortest total trajectory lengths were obtained for the oligotrophic‐to‐mesotrophic VSM, JAB and CER‐L lakes, and longer trajectory lengths were observed for other lakes that reached the eutrophic‐to‐hypereutrophic trophic status (Figure 3B). Lake VSS displayed the longest trajectory, again indicating highest variability in community composition. For each lake, total trajectory length was correlated with the 18‐month averaged Chl*a* concentration (Figure 3C; Table S5) as well as the concentration range (Table S5).

**FIGURE 4:**
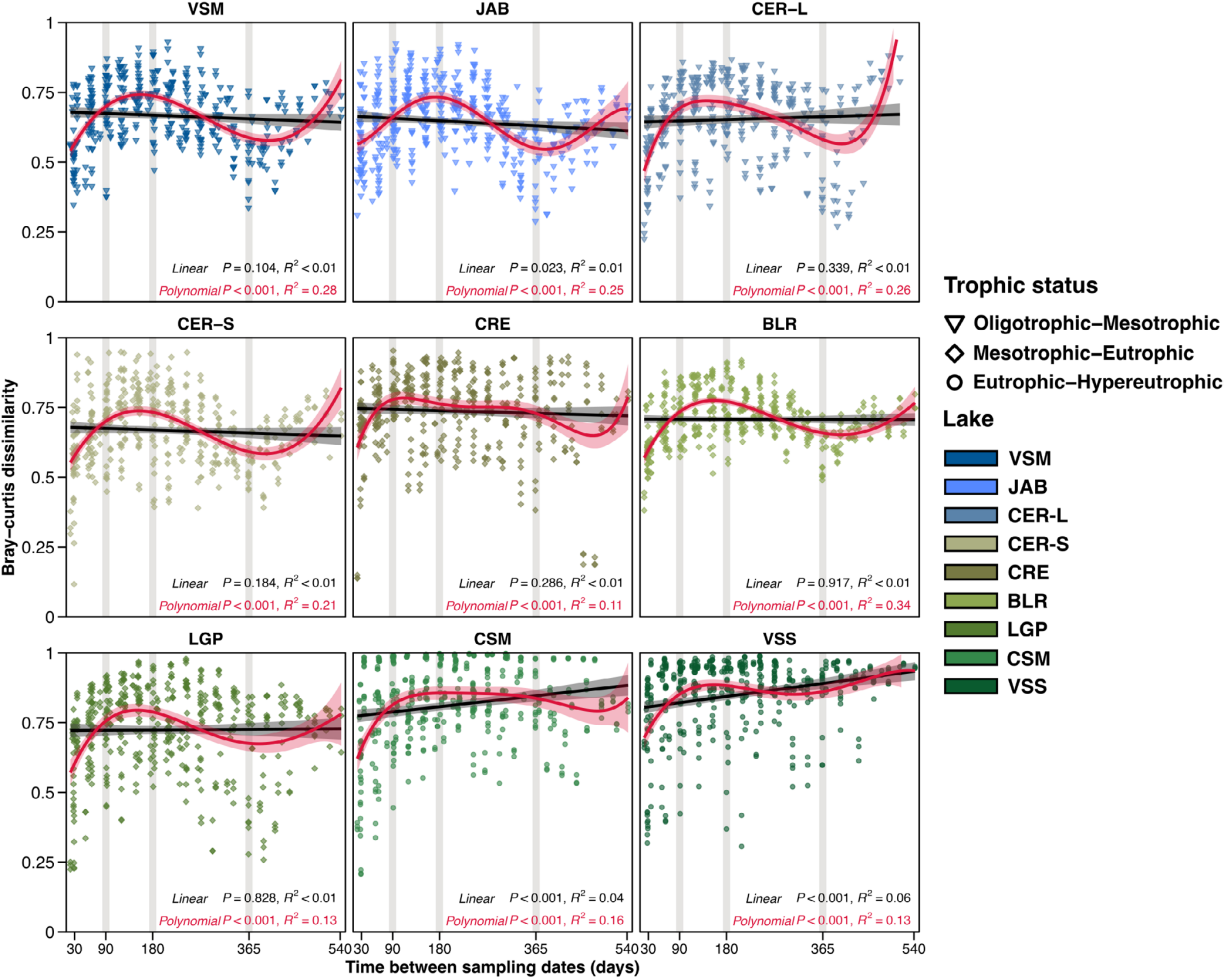
Time‐lag analysis of microeukaryote community dissimilarities. Date‐to‐date Bray‐Curtis dissimilarities computed over all time intervals for each lake (from 22 to 542 days). Linear (black) and polynomial (red) regression are displayed. Significance and rho statistics of each regression are displayed. The 3‐month (90 days), 6‐month (180 days), 12‐month (365 days) time lags are highlighted with vertical grey backgrounds. Lake panels are ordered according to increasing 18‐month averaged Chl*a* concentration (from left to right, then from top to bottom).

Overall similar temporal patterns of microeukaryote community dissimilarities were found in all the lakes based on TLA. The best fit was obtained using polynomial rather than linear models (Figure 4; Table S10). Month‐to‐month dissimilarities were highest when comparing communities approximately 6 months apart (opposite seasons) and lowest for those 1 month apart, emphasising the effect of seasons. When looking at intervals up to 1 year, higher dissimilarities were measured in the eutrophic‐to‐hypereutrophic lakes (CSM and VSS lakes), indicating higher variability in community compositions. Additionally, these two lakes were the only ones for which a linear correlation with a significantly positive slope was found (Figure 4; Table S10). This suggests that these microeukaryote communities did not return to their initial state after 1 year, and diverged with time, exceeding the seasonal oscillations. For the other lakes, non‐significant (*p* > 0.05) or a slightly negative linear regression slope (JAB lake) was observed.

### 3.3 Co‐Occurring Taxa and Network Properties

**FIGURE 5:**
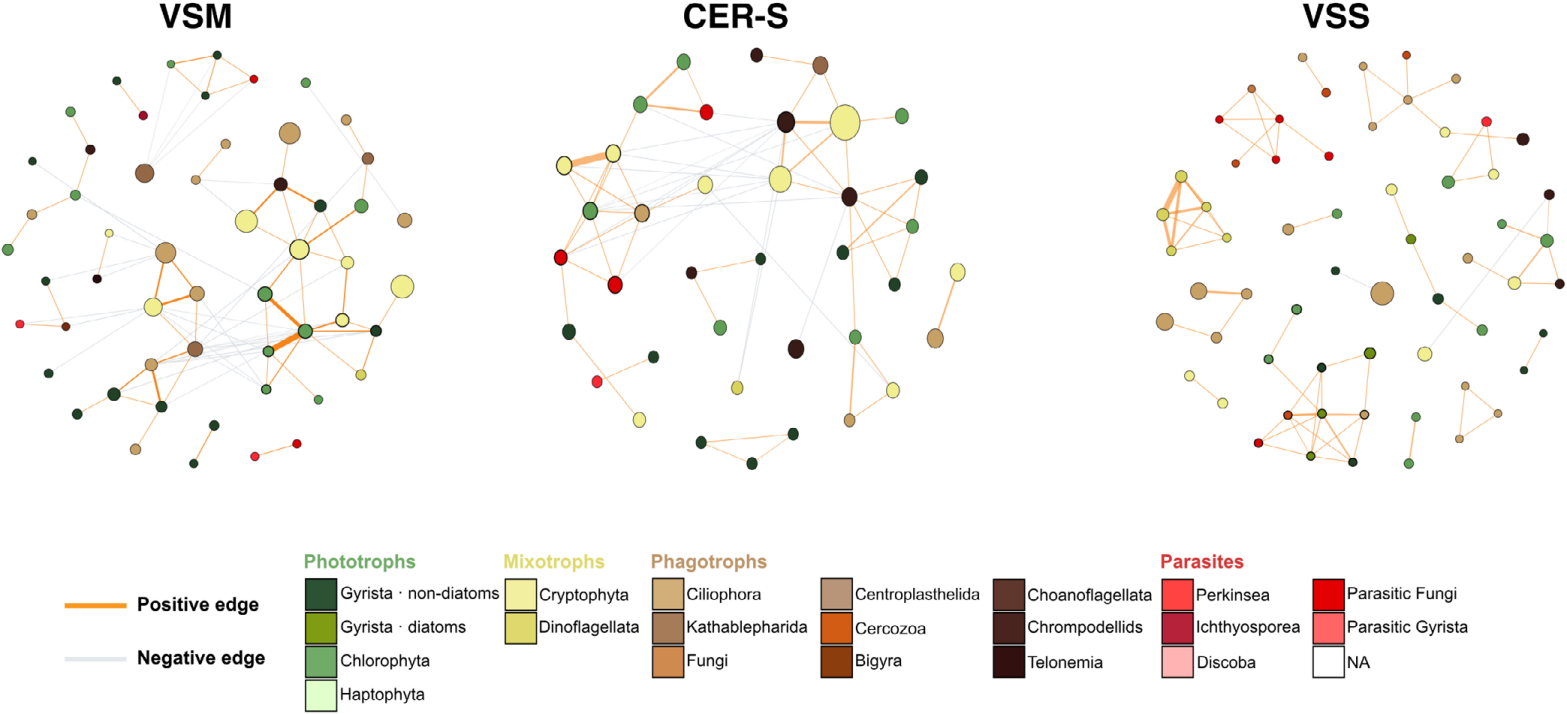
Co‐occurrence networks of the microeukaryote communities. Co‐occurrence networks based on the lake‐specific core ASVs (> 1% of the reads in at least one sample) for lakes VSM (oligotrophic‐to‐mesotrophic), CER‐S (mesotrophic‐to‐eutrophic) and VSS (eutrophic‐to‐hypereutrophic). All networks are displayed in Figure S8. The node diameter indicates mean relative abundance of ASVs and the edge thickness corresponds to the weight of the correlation. The nodes are coloured according to the ASV potential trophic mode (phototrophs, mixotrophs, phagotrophs and parasites, see Figure 2). Lake panels are ordered according to increasing 18‐month averaged Chl*a* concentration (from left to right, then from top to bottom). Only connected nodes are shown.

The number of lake‐specific core ASVs ranged from 126 (JAB and CRE) to 188 ASVs (VSS; Table 1 and S11). The nine networks contained a similar number of connected nodes (52.7 ± 9.7) and modules (i.e., groups of at least two connected nodes; 12.6 ± 2.7). Number of core ASVs, components, clustering coefficient, modularity as well as percentage of positive edges increased with increasing trophic status category from low (VSM, JAB, CER‐L) to intermediate (CER‐S, CRE, BLR and LGP) and high Chl*a* levels (CSM and VSS; Table 1). Trophic richness per module was stable. Overall, these results indicate that lakes of higher trophic status have more structured networks (Figure 5, all networks are displayed in Figure S8), which are likely sensitive to the removal of certain taxa. On the other hand, the networks of the oligotrophic‐to‐mesotrophic lakes (VSM, JAB and CER‐L) displayed a higher number of edges compared to others (Table 1). Moreover, their largest connected component (LCC) contained a higher fraction of the network nodes: 26.8 versus 20.1 and 6.2% for the other two categories (Table 1), indicative of more connected members of the community. Regarding trophic modes, all lakes had comparable percentages of connected nodes affiliated to a given trophic mode: phototrophs (avg. 38.0% ± 7.5%), phagotrophs (33.6% ± 7.6%), mixotrophs (18.7% ± 3.9%), then parasites (9.6% ± 3.1%; Table 1). The network of lake BLR showed notable differences compared to others; it displayed the most connected nodes, largest connected component (LLC, 42.4%), highest percentage of negative edges (39.2%), highest module average trophic richness (2.6) and percentage of modules with the four trophic modes (22.2%), and lowest clustering coefficient (0.41; Figure 5; Table 1), overall indicating more complex trophic interactions.

**TABLE 1:** Principal co‐occurrence network metrics. The complete table is available in Table S11. For each metric, mean average and standard deviation are displayed by trophic status.

Lake	Core ASVs	Components	Edges	Positive edges (%)	Negative edges (%)	Clustering coefficient	Modularity	LCC nodes (%)	Phototrophs connected nodes (%)	Mixotrophs connected nodes (%)	Phagotrophs connected nodes (%)	Parasites connected nodes (%)	Modules with all trophic modes (%)	Module mean trophic richness
VSM	128	82	85	62.35	37.65	0.46	0.50	30.47	42.31	13.46	32.69	11.54	0.00	2.18
JAB	126	98	38	71.05	28.95	0.55	0.57	14.29	34.21	15.79	36.84	13.16	0.00	1.57
CER‐L	137	82	139	70.50	29.50	0.52	0.39	35.77	32.26	17.74	45.16	4.84	8.33	2.17
Oligo‐to‐mesotrophic (mean)	130.3	87.3	87.3	68.0	32.0	0.51	0.48	26.84	36.26	15.66	38.23	9.85	_	1.97
Oligo‐to‐mesotrophic (SD)	5.86	9.24	50.54	4.87	4.87	0.05	0.09	11.19	5.33	2.14	6.35	4.41	_	0.35
CER‐S	132	99	64	73.44	26.56	0.62	0.49	21.21	42.11	23.68	23.68	10.53	11.11	2.33
CRE	126	89	50	96.00	4.00	0.75	0.88	5.56	54.72	13.21	22.64	9.43	0.00	1.71
BLR	151	87	125	60.80	39.20	0.41	0.45	42.38	36.36	22.73	36.36	4.55	22.22	2.56
LGP	167	126	48	85.42	14.58	0.44	0.81	11.38	31.37	19.61	39.22	9.80	0.00	1.64
Mesotrophic‐to‐Eutrophic (mean)	144.00	100.25	71.75	78.91	21.09	0.56	0.66	20.13	41.14	19.81	30.48	8.58	_	2.06
Mesotrophic‐to‐Eutrophic (SD)	18.67	17.95	36.21	15.19	15.19	0.16	0.22	16.18	10.06	4.73	8.54	2.73	_	0.45
CSM	154	112	91	98.90	1.10	0.79	0.74	7.14	37.04	22.22	27.78	12.96	0.00	2.33
VSS	188	142	67	97.01	2.99	0.64	0.86	5.32	31.67	20.00	38.33	10.00	0.00	1.73
Eutrophic‐to‐hypereutrophic (mean)	171.0	127.0	79.0	98.0	2.0	0.71	0.80	6.23	34.35	21.11	33.06	11.48	_	2.03
Eutrophic‐to‐hypereutrophic (SD)	24.04	21.21	16.97	1.33	1.33	0.11	0.08	1.29	3.80	1.57	7.46	2.10	_	0.42
Overall mean	145.4	101.9	78.6	79.5	20.5	0.57	0.6	19.28	38.00	18.72	33.63	9.65	_	2.29
Overall SD	21.5	20.9	34.9	15.1	15.1	0.13	0.2	13.93	7.50	3.92	7.57	3.10	_	2.45

## 4 Discussion

The microeukaryote communities of all the lakes, as estimated by ASVs, were dominated by Cryptophyceae (Cryptophyta), Spirotrichea (Ciliophora), Chrysophyceae (Gyrista) and Chlorophyceae (Chlorophyta). These results support the abundance and importance of mixotrophs and heterotrophs in lakes, and suggest the presence of a common group of generalists, likely phagotrophs (mixotrophs and heterotrophs) year‐round across the geographical area, whatever the trophic status of lakes. In our case, *Cryptomonas curvata* is one such example, being abundant in all lakes and seasons. This is congruent with various studies showing sets of Ciliates, Cryptophyta (including *Cryptomonas*) and Chrysophyceae (including *Dinobryon*) ASVs consistently found year‐round as core members in both temperate and arctic lakes (Potvin et al. 2021; Šimek et al. 2023; Simon et al. 2015). In contrast, no shared set of autotrophs ASVs was identified over the lakes, indicating greater variability within this trophic group, suggesting higher sensitivity to local conditions. However, this finding could be due to the underrepresentation of some taxa in amplicon analysis, as many phototrophic taxa are larger than the size‐fraction fraction used for DNA extraction, including members of the Chlorophyta, Gyrista or Dinoflagellata. Diatoms (Gyrista), were for example much more abundant in the phytoplankton counts than in the 18S rRNA amplicon survey, emphasising that these two types of data cannot be directly compared. Parasitic taxa were present in low abundance in all lakes, as recently found by Garner et al. (2022) in their large survey of Canadian lakes. Parasites may also be underrepresented, due to their association with organisms larger than the size fraction, such as metazoan and larger phytoplanktonic taxa. Additionally, as the primers used are biased against fungi, some taxa like ecologically‐important chytrids that reportedly infect Cyanobacteria (Bower et al. 2004; Gleason et al. 2008; McKindles et al. 2021) and are abundant in temperate lakes such as Lake Sanabria, Spain (Mitsi et al. 2023), may have been overlooked. Despite these possible limitations, lake‐specific patterns were still observed in correlation to the trophic status of these lakes.

### 4.1 Taxa Diversity Is Higher in Lakes of Intermediate Trophic Status

Lower ASV richness was detected for the communities in lakes with both the extreme (highest and lowest) Chl*a* levels compared to lakes with intermediate levels. This is consistent with the Intermediate Disturbance Hypothesis, which predicts diversity peaks at intermediate levels of disturbance or environmental stress, while both low and high extremes tend to favour a reduced set of taxa (Weithoff et al. 2001). Indeed, oligotrophic conditions represent an extreme due to nutrient scarcity, whereas hypereutrophic conditions represent another extreme due to the competitive exclusion exerted by a few dominant taxa. Both conditions select for a more limited number of taxa compared to intermediate conditions (Liu et al. 2019). Recently, lakes of higher trophic status were, for example, reported to display lower diversity of protists in a large‐scale survey (Canada, Garner et al. 2022), as well as in a survey over a more limited region (Ile de France, France, Maloufi et al. 2016; Escalas et al. 2019). In these studies, dominant taxa included bloom‐forming phototrophs as well as various heterotrophs benefiting from high nutrient levels and primary production (Huisman et al. 2018; Garner et al. 2022). On the other hand, intermediate trophic status in a lake may favour a more diverse community by providing more diverse niches for organisms.

### 4.2 Community Structuration and Divergence Are Affected by High Trophic Status

A significant difference in community composition was observed based on the trophic status, confirming its impact on both microeukaryote diversity and composition, especially when comparing the extremes. The maximum difference was found between the oligotrophic‐to‐mesotrophic lakes JAB, VSM, and CER‐L and the eutrophic‐to‐hypereutrophic lakes VSS and CSM. Stronger community structuring in modules was also observed in the networks of VSS and CSM lakes, with each module consisting of different taxa, and limited connectivity among them. Such community structuration suggests that these modules may correspond to taxonomically distinct assemblages potentially associated with different ecological functions, which could imply lower functional redundancy and increased niche compartmentalisation (Xue et al. 2018). On the other hand, increased connectedness in oligotrophic‐to‐mesotrophic lakes suggests a comparatively more complex interaction network and a more stable community, as observed, for example, in studies of bacterial communities (Jiao et al. 2020). Such a pattern would make higher‐trophic‐status lakes communities more vulnerable to functional loss if specific modules are affected by environmental disturbances. In contrast, networks from oligo‐to‐mesotrophic lakes show more connections among taxa and lower modularity, suggesting less niche segregation, reduced sensitivity to taxa loss, and greater functional redundancy. This pattern is consistent with the greater temporal stability observed for these communities. However, it should be noted that such stability occurs under comparatively less variable environmental conditions in these lakes. Thus, it does not necessarily imply that these communities would be better able to cope with eventual disturbances than those of lakes VSS and CSM.

Lake BLR appears as an outlier compared to others. Despite its mesotrophic‐to‐eutrophic status, it displays stable microeukaryote community composition. Its associated network is the most diffuse, with the highest number of connections, lowest clustering coefficient, and highest number of trophic modes per module indicating stable communities with a complex network of interactions. Its phytoplanktonic community is also dominated by Cyanobacteria. In a previous study, prokaryotic communities were also found to be highly stable in lake BLR and different from other lakes (Foucault et al. 2025) suggesting that peculiar local conditions shape both eukaryotic and prokaryotic communities. None of the parameters examined in the present study explained the difference with other lakes, indicating that trophic status alone is insufficient to explain observed temporal dynamics. Local peculiarities (pedology, type of vegetation, land use, presence of unmeasured contaminants) should be further explored to explain the difference between this and the eight other lakes.

### 4.3 Stable Temporal Patterns Are Detected but Hypereutrophic Status Induce Community Shifts

Overall similar temporal patterns are observed in all lakes, congruent with our first hypothesis, with a summer alpha diversity (richness) being around twice that observed in winter, which is not surprising given their close geographical proximity. This trend holds true for all microeukaryotes trophic modes (phototrophs, mixotrophs, phagotrophs and parasites), indicating comparable response. Higher eukaryote diversity during summer and autumn has often been observed in temperate lakes, as evidenced in the 20 years' time‐series conducted in Lake Mendota (Krinos et al. 2024). Within a one‐year interval, community compositions are most different in opposite seasons (winter versus summer, for example), as expected under temperate climates (Simon et al. 2015; David et al. 2021). They tend to be more similar after 1 year, indicating cyclic variations in community structure, as regularly documented in longer time series (David et al. 2021; Krinos et al. 2024). However, despite overall similar trends, lakes that reach the hypereutrophic status (CSM and VSS) displayed unique features. First, higher variability of microeukaryote communities was observed throughout the 18‐month period, not limited to the maximal primary productivity period (i.e., summer), with higher month‐to‐month dissimilarities compared to other lakes. This is congruent with the hypothesis formulated by Garner et al. (2022), based on their Canadian survey, that protist communities in productive lakes are less stable over time. Second, community compositions in CSM and VSS (eutrophic‐to‐hypereutrophic) lakes also tend not to return to their initial state after 1 year, showing signs of year‐to‐year divergence, in a way similar to that observed by David et al. (2021) in microeukaryote communities over a two‐year time series on a set of small ponds. This trend is not observed in lakes displaying lower trophic status, suggesting that hyper‐eutrophication may ultimately lead to increased community composition drift with time. The hypothesis that community variability will decrease under higher trophic status is thus not supported in this study. The hypothesis of a regime shift in the same hypereutrophic CSM and VSS lakes was recently formulated for prokaryotic communities investigated during the summer of 2021 (Foucault et al. 2025). In the present time‐series, it must be noted that lake CSM reached the hypereutrophic status during the summer 2021, then shifted to oligotrophic status afterwards, possibly also explaining community changes. Yet, a recent 120‐years survey, authors found that re‐oligotrophication of eutrophicated lakes did not lead to a return of communities to a pre‐disturbance state (Barouillet et al. 2025). Our results overall suggest that microeukaryote communities in lakes reaching the hypereutrophic status start diverging with time. Whether hypereutrophic conditions represent a tipping point beyond which the divergence of microeukaryote communities increases over time will require additional testing for year‐to‐year drift over a period of several years.

## 5 Conclusion

This study shows that the eutrophication level has an impact on microeukaryote community composition, and that this variability is particularly high in lakes reaching the hypereutrophic status. Diversity was maximal at intermediate trophic status, congruent with the Intermediate Disturbance Hypothesis. Mixotrophic and heterotrophic taxa dominate, with a few taxa being abundant in all lakes year‐round. Lakes reaching the hypereutrophic status show markedly increased variations throughout all seasons and potential year‐to‐year drift in community compositions compared to other lakes. Long‐term monitoring of lakes spread over a limited area, such as the Ile de France region, would provide an opportunity to test the hypothesis of a regime shift and identify the exact conditions that trigger it.

## Author Contributions

**Sébastien Duperron:** conceptualization, investigation, funding acquisition, writing – original draft, project administration, supervision. **Emilie Lance:** investigation. **Amaury Le Vern:** data curation, formal analysis. **Benjamin Marie:** conceptualization, investigation, writing – review and editing. **Midoli Goto:** investigation. **Pierre Foucault:** investigation, data curation, writing – original draft, formal analysis. **Charlotte Duval:** investigation. **Cécile Bernard:** conceptualization, investigation, writing – review and editing. **Sébastien Halary:** conceptualization, investigation, writing – review and editing. **Sahima Hamlaoui:** conceptualization, investigation, writing – review and editing. **Ludwig Jardillier:** conceptualization, investigation, funding acquisition, supervision, writing – original draft, project administration. **Marc Troussellier:** conceptualization, investigation, writing – review and editing. **Dominique Lamy:** investigation. **Julie Leloup:** conceptualization, investigation, funding acquisition, writing – original draft, project administration, supervision.

## Funding

This work was supported by Agence Nationale de la Recherche, ANR‐20‐CE32‐0006.

## Conflicts of Interest

The authors declare no conflicts of interest.

## Supporting information


**Figure S1:** Location of the lakes within the Île‐de‐France region (France).
**Figure S2:** Analysis of physico‐chemical parameters.
**Figure S3:** Temporal dynamics of the phytoplankton community composition.
**Figure S4:** Taxonomic composition of microeukaryote community composition.
**Figure S5:** Temporal variation of the diversity indexes of microeukaryote communities.
**Figure S6:** Microeukaryote community composition for each individual lake.
**Figure S7:** Microeukaryote communities structure based on trophic modes.
**Figure S8:** Co‐occurrence networks of the microeukaryote communities.

**Table S1:** Samples nomenclature, SRA accession numbers and main bioinformatic metrics.
**Table S2:** Chla concentrations (μg·L−1).
**Table S3:** Physico‐chemical parameters.
**Table S4:** Microeukaryote taxonomy.
**Table S5:** Summary of Spearman's rank tests.
**Table S6:** Phytoplankton taxonomy.
**Table S7:** Relative abundance by lake and season of microeukaryote taxa.
**Table S8:** Phytoplankton taxonomy.
**Table S9:** Summary of permanova tests.
**Table S10:** Summary of linear and polynomial model regressions.
**Table S11:** Summary of network properties.

## Data Availability

Raw reads were deposited into Sequence Read Archive (SRA, Project PRJNA1086840, see Table S1 for sample accession numbers). Scripts are available at https://github.com/PierreFoucault/Greater‐Paris‐microEukaryotes.

## Data Availability Statement

All 18S rRNA gene amplicon sequencing raw reads were deposited into the Sequence Read Archive (SRA) database under the BioProject PRJNA1086840 (see Table S1 for individual sample SRA accession numbers). Scripts available at https://github.com/PierreFoucault/Greater‐Paris‐microEukaryotes.
