# Supplementary material for: Higher Trophic Status Leads to More Diverse and Divergent Microeukaryote Communities Over Time in Urban Lakes From the Greater Paris (France)
Source: Environ Microbiol Rep. 2026 Apr 28;18(3):e70355. doi: 10.1111/1758-2229.70355 (PMC13124647; doi:10.1111/1758-2229.70355)

**Higher trophic status leads to more diverse and divergent microeukaryote communities over time in urban lakes from the Greater Paris (France)**

Sébastien Duperron^1^*, Pierre Foucault^1,2^, Amaury Le Vern^1^, Midoli Goto^1,3^, Charlotte Duval^1^, Benjamin Marie^1^, Sahima Hamlaoui^1^, Sébastien Halary^1^, Dominique Lamy^2^, Emilie Lance^1,5^, Marc Trousselier^3^, Cécile Bernard^1^, Ludwig Jardillier^4^, Julie Leloup^2^*

**Supplemental data**

**Table S1: Samples nomenclature, SRA accession numbers and main bioinformatic metrics**

**Table S2: Chla concentrations (µg.L^-1^)**

**Table S3: Physico-chemical parameters**

**Table S4: Microeukaryote taxonomy**

**Table S5: Summary of Spearman's rank tests**

**Table S6: Phytoplankton taxonomy**

**Table S7: Relative abundance by lake and season of microeukaryote taxa**

**Table S8: Phytoplankton taxonomy**

**Table S9: Summary of permanova tests**

**Table S10: Summary of linear and polynomial model regressions**

**Table S11: Summary of network properties**

**Fig. S1: Location of the lakes within the Île-de-France region (France)**

**Fig. S2: Analysis of physico-chemical parameters**

**Fig. S3: Temporal dynamics of the phytoplankton community composition**

**Fig. S4: Taxonomic composition of microeukaryote community** **composition**

**Fig. S5: Temporal variation of the diversity indexes of microeukaryote communities**

**Fig. S6: Microeukaryote community composition for each individual lake**

**Fig. S7 Microeukaryote communities structure based on trophic modes**

**Fig. S8: Co-occurrence networks of the microeukaryote communities**

**Fig. S1: Location of the lakes within the Île-de-France region (France).**

Lakes identifiers are: Jablines (JAB), Vaires-sur Marne (VSM), Cergy large (CER-L), Cergy small (CER-S), Créteil (CRE), Bois-le-Roi (BLR), La Grande Paroisse (LGP), Champs-sur-Marne (CSM), Verneuil-sur-Seine (VSS).
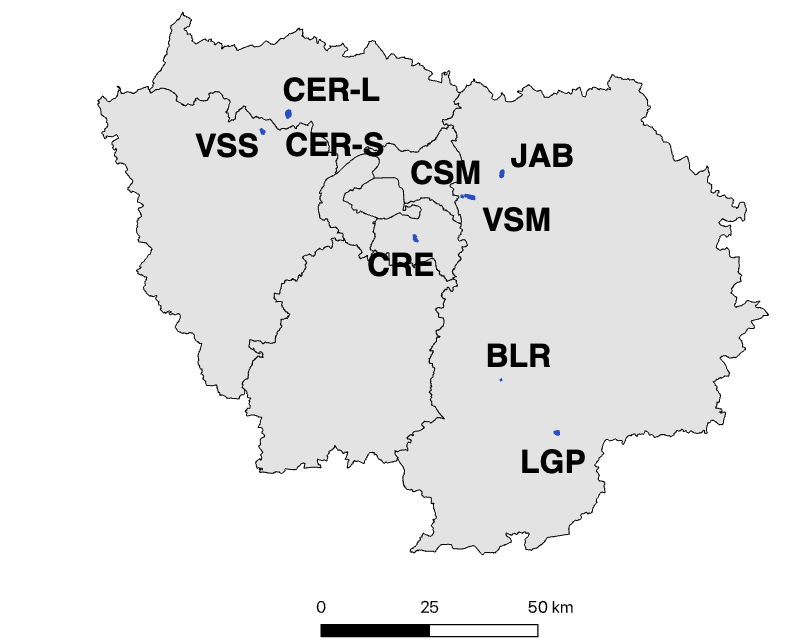
GPS coordinates are in Table S1.

**Fig. S2: Analysis of physico-chemical parameters**

PCA based on physico-chemical parameters (T, pH, TPC, TPN, PO_4_^3-^, NH_4_^+^, NO_3_^-^+NO_2_^-^). Lakes are displayed in individual panels based on the same coordinates set (indicated by identical axis and grey points) and ordered according to their 18-month averaged Chl*a* concentration (from left to right, then from top to bottom). Seasons are colored and delimited by polygons representing the maximal area delimited by the sample’s coordinates.

**Fig. S3: Temporal dynamic of the phytoplankton community composition**

Relative biovolume (biovolume-adjusted cell count) of the phytoplankton taxa for each lake as percentage of the total phytoplankton biovolume. Lake panels are ordered according to increasing 18-month averaged Chl*a* concentration (from left to right, then from top to bottom). The x-axis corresponds to the month (*i.e.,* initial of each month) and the color bars correspond to seasons.


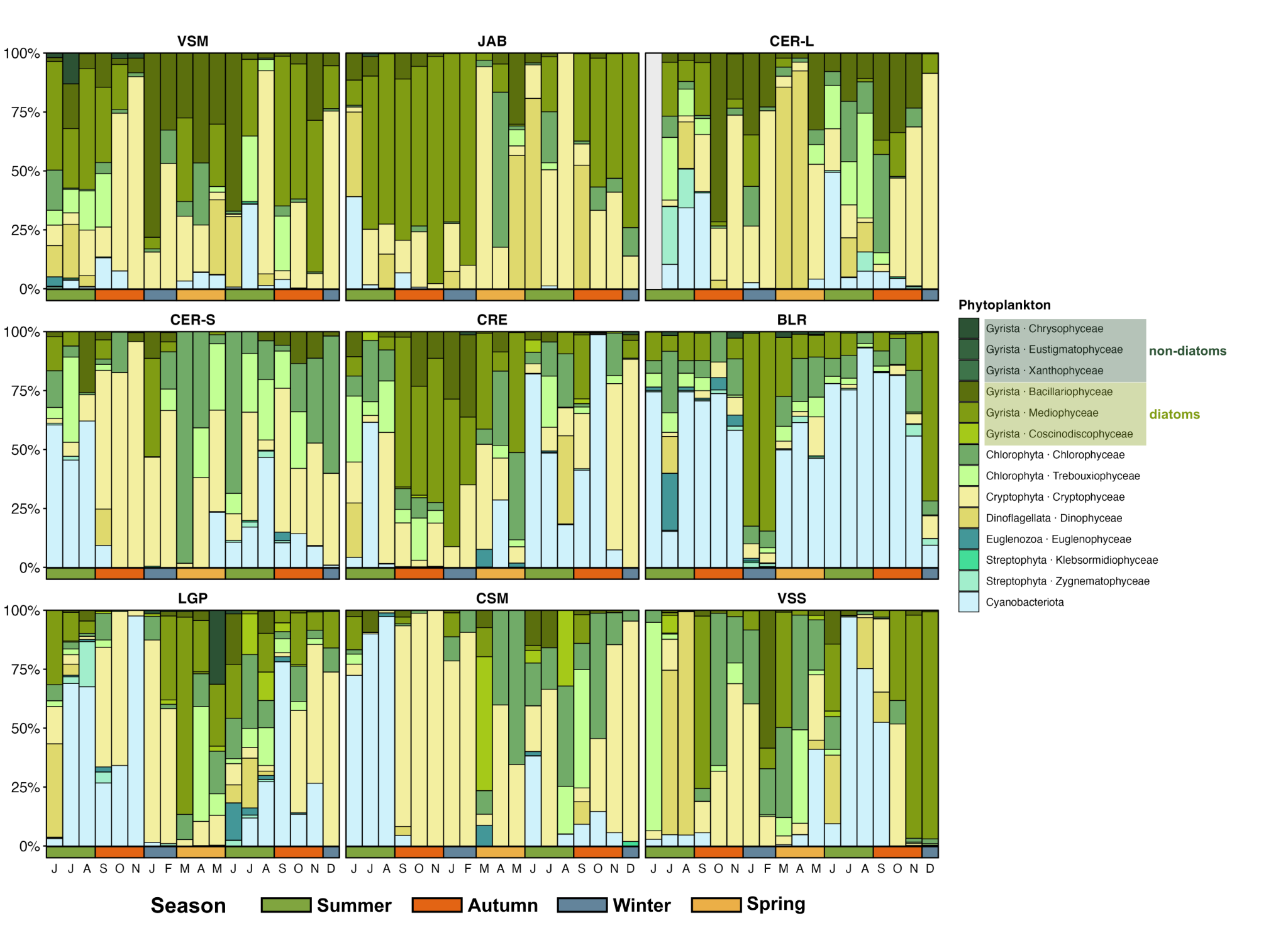


**Fig. S4: Taxonomic composition of microeukaryote community** **composition**

Data are presented as median proportion of total ASV reads per month. The 25 most abundant classes are colored according to their potential trophic mode (phototrophs, mixotrophs, phagotrophs and parasites). On the x-axis, the letters correspond to the months (June 2021 to December 2022) and the color bars to the seasons. Lake panels are ordered according to increasing 18-month averaged Chl*a* concentration (from left to right, then from top to bottom).


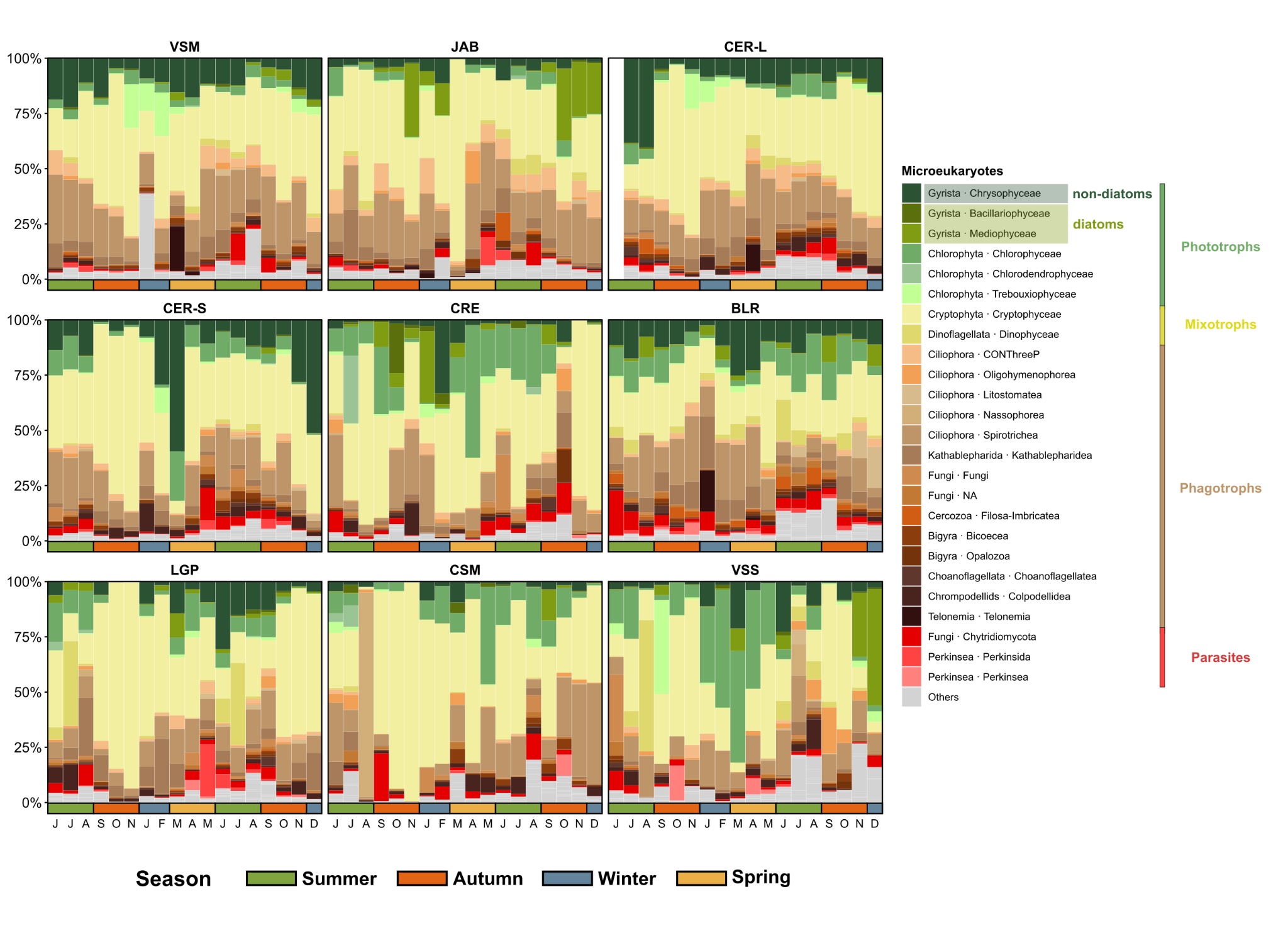


**Fig. S5: Temporal variation of the diversity indexes of microeukaryote communities**

**A-B:** Richness and Shannon diversity index over the 18 months (540 days) for the total ASVs. **C-F:** Richness of ASVs assigned to phototrophs (**C**), mixotrophs (**D**), phagotrophs (**E**), and parasites (**F**).


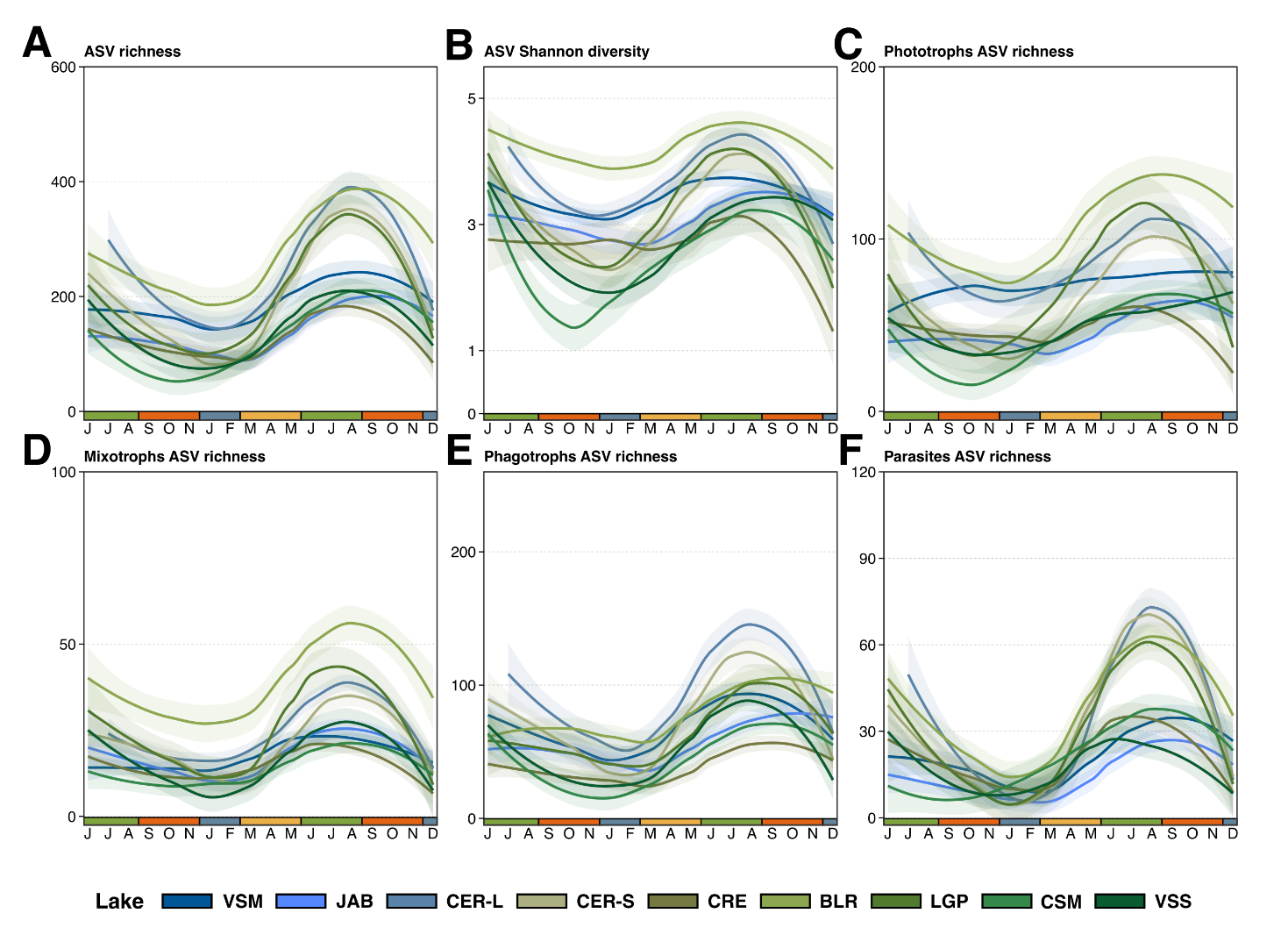


**Fig. S6: Microeukaryote community composition for each individual lake**

PCoA plot based on Bray-Curtis dissimilarity of microeukaryote communities. Lakes communities are displayed in individual panels based on the same coordinates set (indicated by identical axis and grey points) and ordered according to their 18-month averaged Chl*a* concentration (from left to right, then from top to bottom). Seasons are colored and delimited by polygons representing the maximal area delimited by the sample’s coordinates.

**
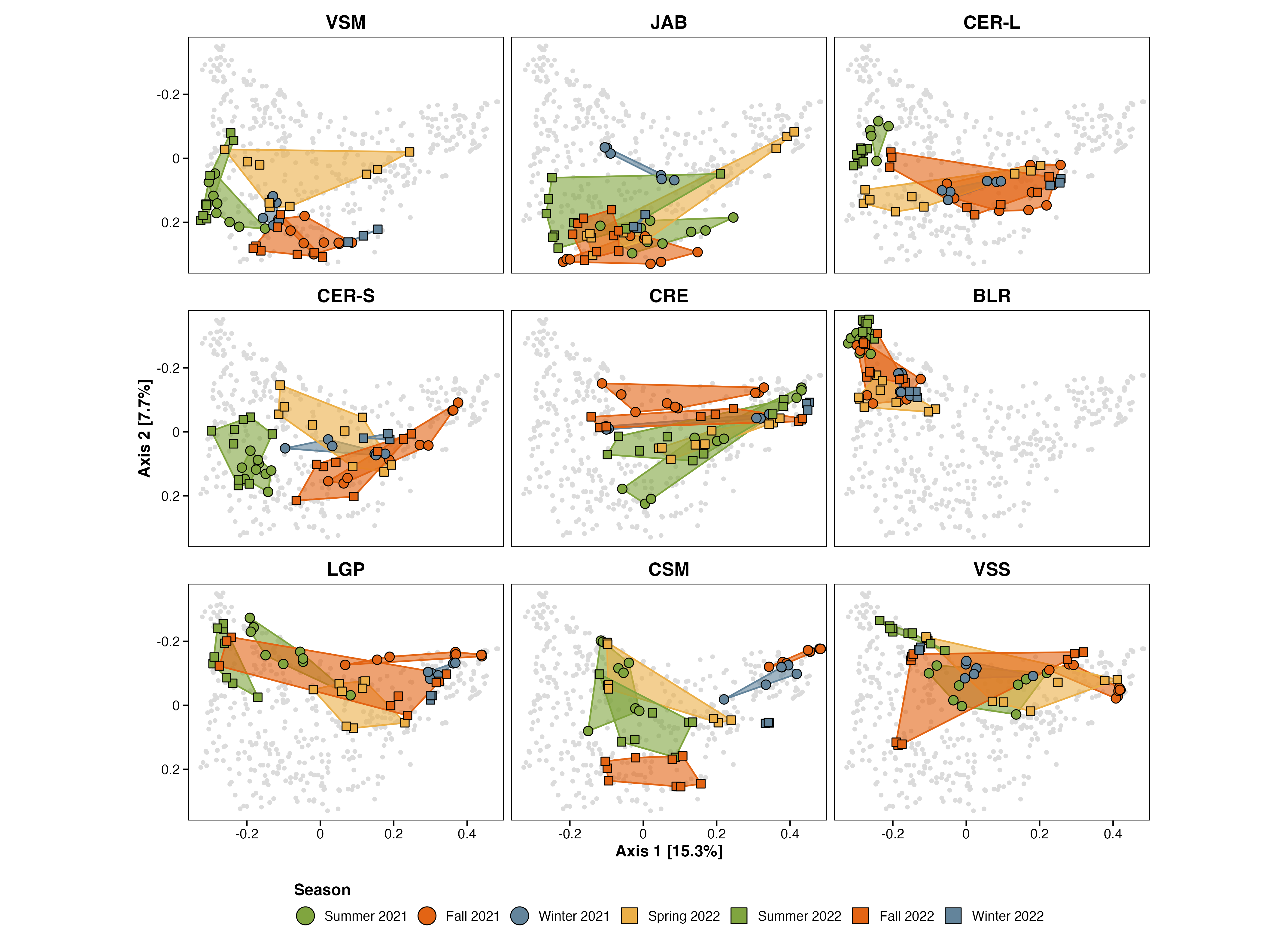
**

**Fig. S7: Microeukaryote communities structure based on trophic modes**

PCoA plots (based on Bray-Curtis dissimilarity) based on ASVs classified as phototrophs (**A**), mixotrophs (**B**), phagotroph (**C**) and parasites (**D**). Lakes are displayed in individual panels based on the same coordinates set (indicated by identical axis and grey points) and ordered according to their 18-month averaged Chl*a* concentration (from left to right, then from top to bottom). Seasons are colored and delimited by polygons representing the maximal area delimited by the sample’s coordinates.


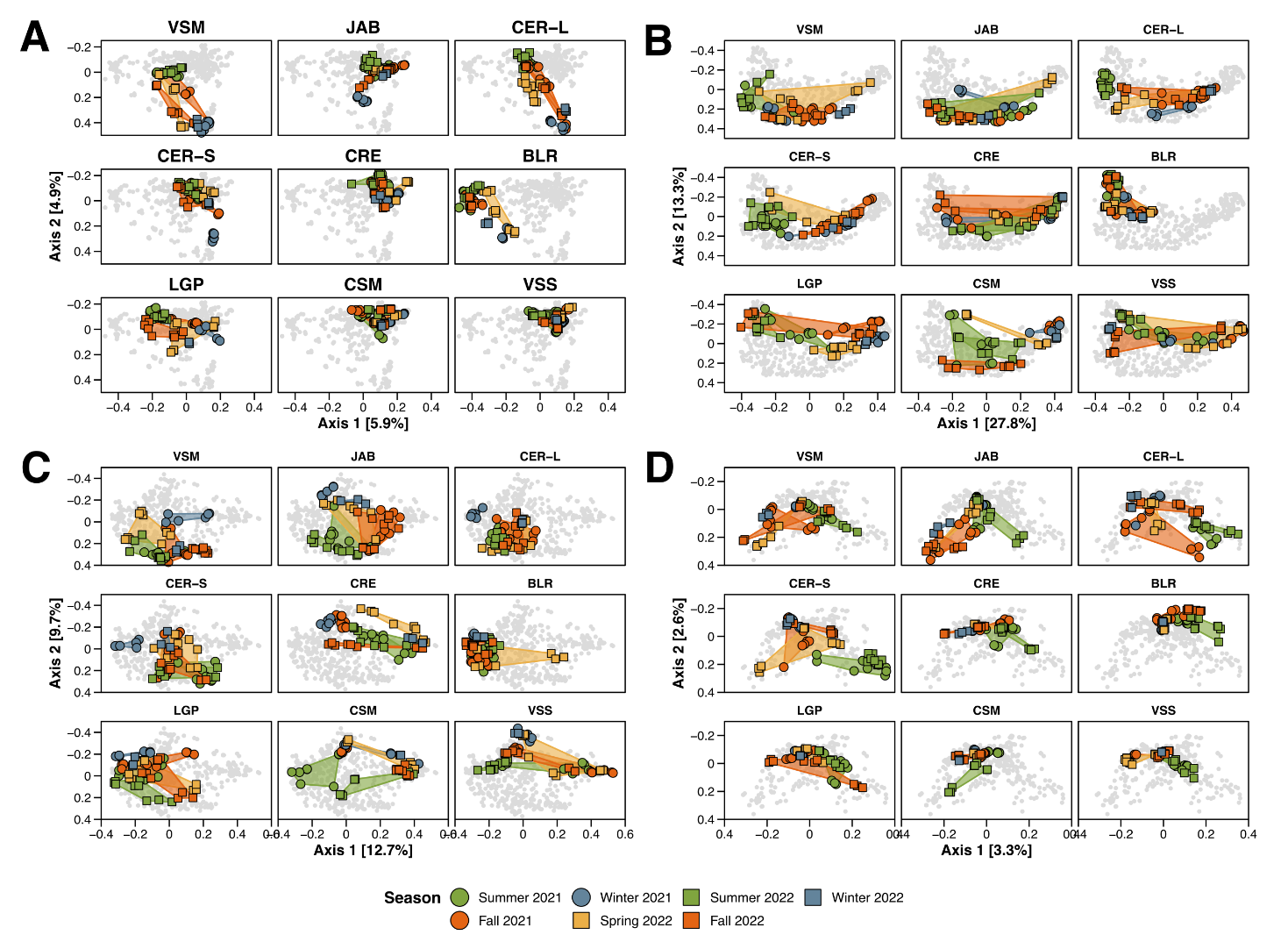


**Fig. S8: Co-occurrence networks of the microeukaryote communities**

Co-occurrence networks based on the lake-specific core ASVs (>1% of the reads in at least one sample). The node diameter indicates mean relative abundance of ASVs and the edge thickness corresponds to the weight of the correlation. The nodes are colored according to the ASV potential trophic mode (phototrophs, mixotrophs, phagotrophs and parasites, see Fig. 2). Lake panels are ordered according to increasing 18-month averaged Chl*a* concentration (from left to right, then from top to bottom). Only connected nodes are shown.


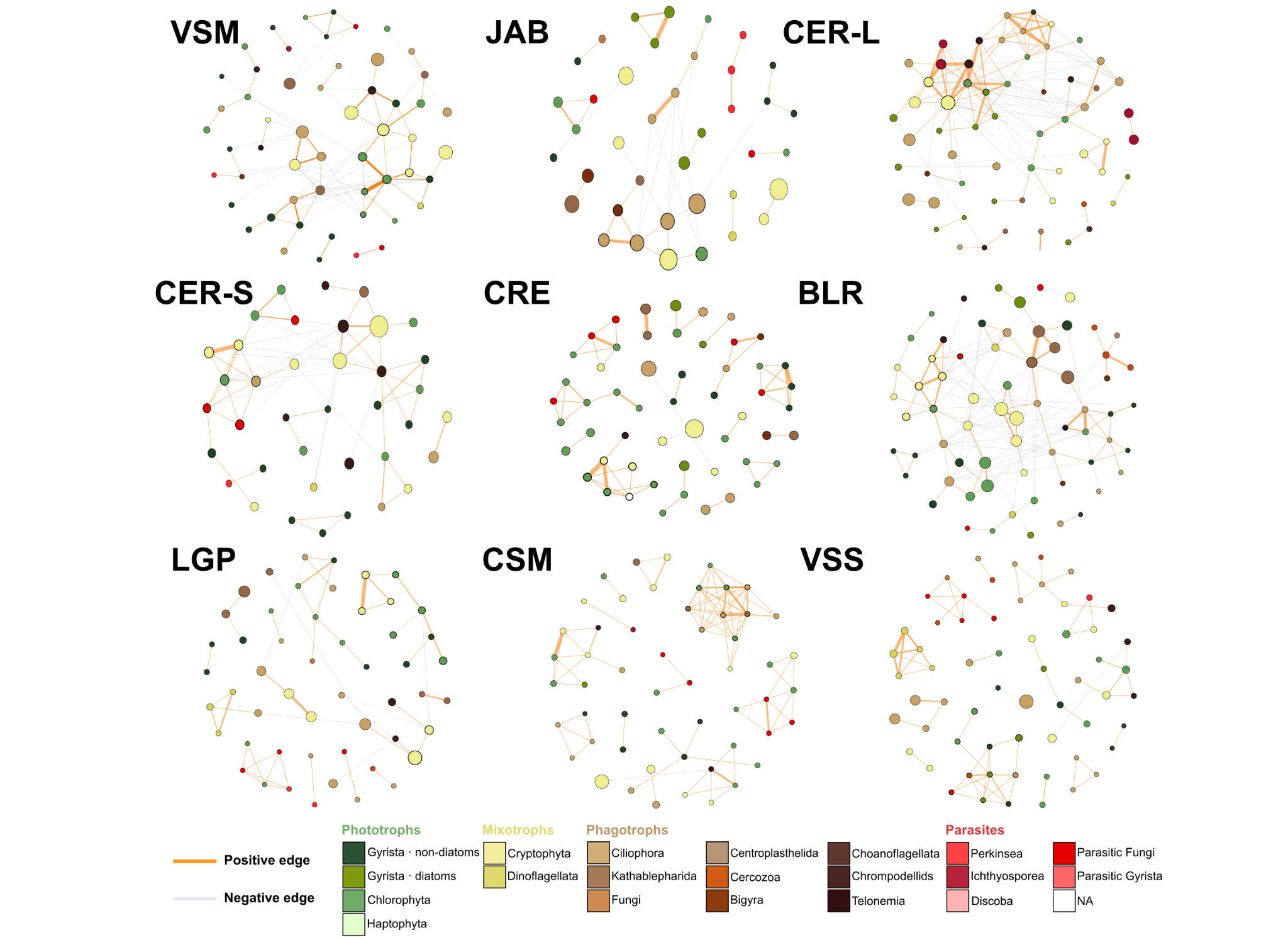

Supplement: Supplementary file 1 — Figure S1: Location of the lakes within the Île‐de‐France region (France). Figure S2: Analysis of physico‐chemical parameters. Figure S3: Temporal dynamics of the phytoplankton community composition. Figure S4: Taxonomic composition of microeukaryote community composition. Figure S5: Temporal variation of the diversity indexes of microeukaryote communities. Figure S6: Microeukaryote community composition for each individual lake. Figure S7: Microeukaryote communities structure based on trophic modes. Figure S8: Co‐occurrence networks of the microeukaryote communities. [file EMI4-18-e70355-s002.docx]
